# Self-reported hearing difficulties, main income sources, and socio-economic status; a cross-sectional population-based study in Sweden

**DOI:** 10.1186/1471-2458-12-874

**Published:** 2012-10-15

**Authors:** Pernilla Videhult Pierre, Anders Fridberger, Anders Wikman, Kristina Alexanderson

**Affiliations:** 1 Karolinska Institutet, Center for Hearing and Communication Research, Department of Clinical Science, Intervention, and Technology, Karolinska University Hospital, Stockholm, Sweden; 2 Division of Insurance Medicine, Department of Clinical Neuroscience, Karolinska Institutet, Stockholm, Sweden

**Keywords:** Disability pension, Hearing loss, Occupation, Self-reported health, Sick leave, Socio-demographic factors, Socio-economic status, Unemployment, Working

## Abstract

**Background:**

Hearing difficulties constitute the most common cause of disability globally. Yet, studies on people with hearing difficulties regarding socio-economic status (SES), work, long-term unemployment, sickness absence, and disability pension are scarce. The aim of the present study was to investigate the main income sources of men and women of working ages with and without self-reported hearing difficulties and associations with gender, age, SES, type of living area, and country of birth.

**Methods:**

A cross-sectional population-based study, using information on self-reported hearing difficulties and SES of 19 045 subjects aged 20–64 years participating in Statistics Sweden’s annual Living Conditions Surveys in any of the years 2004 through 2008. The information was linked to a nationwide database containing data on demographics and income sources. Odds ratios (ORs) and their 95% confidence intervals (CIs) were calculated, using binary logistic regression analysis.

**Results:**

Hearing difficulties increased with age and were more common in men (age-adjusted OR: 1.42 (95% CI: 1.30-1.56)) with an overall prevalence of 13.1% in men and 9.8% in women. Using working men as reference, the OR of having hearing difficulties was 1.23 (0.94-1.60) in men with unemployment benefits and 1.36 (1.13-1.65) in men with sickness benefits or disability pension, when adjusting for age and SES. The corresponding figures in women were 1.59 (1.17-2.16) and 1.73 (1.46-2.06). The OR of having sickness benefits or disability pension in subjects with hearing difficulties was 1.36 (1.12-1.64) in men and 1.70 (1.43-2.01) in women, when adjusting for age and SES and using men and women with no hearing difficulties as reference.

**Conclusions:**

Hearing difficulties were more prevalent in men. After adjustment with age and SES as well as with type of living area and country of birth, a significant association with unemployment benefits was found only in women, and the associations with long-term sickness absence and disability pension tended to be stronger in women.

## Background

Hearing difficulties are the most common cause of disability globally [1]. Since they are highly age-related, their global impact on burden of disease is expected to grow due to the increasing life span [1]. Studies suggesting that the age-specific prevalence is on the rise as well is of great concern [2,3]. In Sweden, the proportion of people aged 16–84 years with self-reported hearing difficulties has increased from 10.5% in 1984–1985 to 14% 20 years later [3]. This negative development is particularly pronounced in young people, in agreement with recent audiometric investigations [4,5], and in women [3]. Why the age-specific prevalence would be increasing is unknown, but potential contributing factors are use of personal listening devices [6-8], diabetes [9,10], cardiovascular disease [11,12], common analgesic drugs [13], distress [14,15], and higher demands of communication skills in modern society, which possibly has led to a higher awareness of hearing difficulties [16]. However, other studies indicate that the age-specific prevalence is instead decreasing [17-19], possibly as a result of better medical management of middle-ear disease in childhood [20] and a reduction in occupational noise-induced hearing loss due to the introduction of hearing conservation programs and a decrease of total employment in manufacturing [21]. Differences in hearing assessment methods and in subjects included may partly explain the discordant results.

Several methods are used for hearing assessment, the most common being pure tone audiometry (PTA) and self-reported hearing measures. PTA relies on patient response to pure tone stimuli and is the most frequent method of clinical hearing assessment. It measures ear-specific hearing thresholds rather than other aspects of hearing, such as sound localization, and the auditory situation is not very similar to normal listening. Self-reported hearing measures are particularly useful for surveying hearing difficulties in a large population since they are easy to administer. The correlations between PTA and self-reported hearing measures have been investigated (e.g. [22-24]). As expected, pure-tone thresholds in the speech frequency range are more closely related to self-reported hearing difficulties than is high-frequency PTA [22]. However, the correlation is also dependent on other factors, such as age [22,23], severity of hearing threshold elevations [22,24], and prevalence of other somatic symptoms [23]. Thus, while some individulals with self-reported hearing difficulties show no PTA-assessed hearing threshold elevations, others without such self-reported difficulties show significant PTA-assessed hearing threshold elevations [23].

People with hearing difficulties constitute a vulnerable group in society. They are at increased risk of underperforming in school [25-27], of being underrepresented in highly skilled jobs [28], and of being overrepresented among low-income earners [2,26,28] and unemployed [25,26,28,29]. Working hearing-impaired individuals often experience less control over their work environment, which may eventually result in stress-related health problems [30,31]. Several studies have shown associations between hearing difficulties and long-term health problems [14,32], sickness absence [30,32], and disability pension [25,33,34].

Manual workers are overrepresented among those with hearing difficulties [35-37], an important reason being occupational noise exposure [38]. Although socio-economic status (SES) is highly correlated with unemployment and health [39], studies on hearing difficulties, unemployment, and sickness absence that adjust for SES are scarce [40]. In a recent register-based study in Sweden, an increased risk of future disability pension was found among individuals with sickness absence due to a hearing diagnosis compared to those with non-otoaudiological sick leave diagnoses after adjustment for a wide range of potential confounders, such as age, sex, family situation, type of living area, birth region, years of education, and hospitalization days [33].

In general, there is a lack of knowledge on SES and type of income of people with self-reported hearing difficulties, compared to others. The aim of the present study was to investigate the main income sources of men and women of working ages with and without self-reported hearing difficulties and associations with gender, age, SES, type of living area, and country of birth.

## Methods

### Study population and data

A cross-sectional population-based study was conducted, using survey and register data from Sweden. Included subjects (n=19 045) had participated in the Living Conditions Surveys (ULF/SILC (Undersökningarna av levnadsförhållanden/Statistics on Income and Living Conditions; more details are given below)) in any of the years 2004 through 2008 and were registered as living in Sweden in 2005, according to Statistics Sweden’s database LISA (Longitudinal integration database for health insurance and labor market studies). They were aged 20–64 years the year of participating in ULF/SILC, were not on early old-age pension, and had answered “yes” or “no” on the ULF/SILC question about having hearing difficulties. Only five subjects had failed to give this answer. None had participated more than once in ULF/SILC during these years. Information from ULF/SILC for these subjects was linked to the LISA database for the same year, using the unique personal identification number assigned to all residents in Sweden.

The ULF survey is conducted yearly since 1975 on a random sample of people living in Sweden. Until 2006, data collection was mainly performed through face-to-face interviews, but after this telephone interviews are the main method of data collection. In 2008, the survey European Union Statistics on Income and Living Conditions (EU-SILC) was integrated with ULF, forming ULF/SILC. The response rate in 2004 through 2008 was about 75% [41].

LISA contains information on demographics and income from work, social security sources, and student allowance of all individuals above 15 years of age, registered as living in Sweden as of December 31 of each year [42]. As further described in **Factors included in the analyses**, each included subject was assigned to one of several main income source categories based on their gross income source and number of days of gross income during the year of participation in ULF/SILC.

### Social insurance in Sweden

All individuals in Sweden with income from work or unemployment benefits are covered by the same public sickness insurance. The Social Insurance Agency provides sickness benefits to people who cannot work due to sickness. The employer provides sick pay the first 14 days of a sick-leave spell for employees. If the work capacity is permanently reduced, disability pension can be granted. Sickness benefits cover about 80% and disability pension at least 65% of the lost income [43]. The retirement age is generally 65 years but can be taken earlier.

Parents can be off work with parental benefits for 480 days per child. The benefits cover about 80% of the lost income for 390 days and less for the remaining 90 days [44].

### The Swedish financial aid for studies

Students in Sweden can apply for government grants and loans (here called student allowance) to cover costs of living when attending university or adult secondary education programs (there are no student fees for Swedish citizens). In 2012, full-time students can be granted student allowance of a maximum of 44,600 SEK (~5,000 EUR) per semester [45].

### Factors included in the analyses

#### Self-reported hearing difficulties

Self-reported hearing difficulties were assessed using the question on hearing difficulties in ULF/SILC. In 2004 through 2007, the question “Can you without difficulties hear what is said in a conversation between several people with or without a hearing aid?” was used. In 2008, the question “Do you have difficulties hearing what is said in a conversation between several people, even if using a hearing aid?” was used. Response alternatives were “yes” and “no” [41].

#### Main income source

Using LISA data, the subjects were assigned to one of eight different categories of main income source: A, work; B, parental benefits; C, student allowance; D, social assistance; E, unemployment benefits; F, sickness benefits; G, disability pension; H, miscellaneous. The main income source of a subject was defined as the most prevalent one the year of participation in ULF/SILC. A subject was assigned to category A (work) if his/her income from work exceeded a minimum amount, predefined by Statistics Sweden, where they had used an advanced regression model to render data consistent with other statistics [46]. If having parental benefits, unemployment benefits, sickness benefits, or disability pension for at least six months that year, a subject was instead assigned to one of those categories (B, E, F, or G, respectively). A subject was assigned to category C (student allowance) or D (social assistance) if more than half of the annual income consisted of such compensation, as information on number of days with such income was not available. The remaining subjects (e.g. homemakers, students without allowance, and unemployed without benefits) were assigned to category H (miscellaneous). The categories were mutually exclusive.

#### Age

Age was used as a continuous variable or categorized into five strata, 20–24, 25–34, 35–44, 45–54, and 55–64 years.

#### SES

Based on ULF/SILC data, the subjects were classified into the following SES categories, using the socio-economic classification system of Statistics Sweden [41,47]: professional work level (employed and self-employed, including other higher non-manual employees, with or without subordinates), intermediate non-manual work level, assistant non-manual work level, self-employed non-professional work level (including farmers), skilled manual work level, unskilled/semiskilled manual work level, and students. Working and non-working subjects were classified mainly according to their present and previous occupation, respectively. Until 2007, homemakers were assigned to the same SES category as their wife/husband. People not working but studying ≥ 16 hours per week were classified as students. In 2008, homemakers and those studying < 16 hours per week were instead classified according to their previous occupation or, if no previous occupation, his/her educational level. Individuals studying ≥ 16 hours per week were classified as students, but if working > 16 hours per week, classification was instead based on that specific job. However, in 2008, all individuals below 22 years of age were classified as students, even if working > 16 hours per week. Since the changes in 2008 have influenced the distribution of the SES categories only slightly [41], their impact on the results of the present study are likely insignificant.

#### Type of living area

Resident municipality data from LISA were used to classify the subjects as living in urban (including Stockholm (H1), Gothenburg, and Malmö (H2)), semi-urban (H3; municipalities with more than 90,000 inhabitants), or sparsely populated (H4-H6) areas, based on Statistics Sweden’s homogenous region (H region) classification system [48].

#### Country of birth

Information on country of birth was obtained from LISA and was dichotomized into Sweden and elsewhere.

### Statistical analyses

Different types of associations were calculated with either having hearing difficulties or having sickness benefits or disability pension as outcome measure.

In analyses where hearing difficulties were used as outcome measure, the eight categories of main income source were reduced to four in order to obtain sufficient statistical power (work (A), unemployment benefits (E), sickness benefits or disability pension (F+G), and others (B+C+D+H)). For the same reason, the eight categories were dichotomized into having sickness benefits or disability pension (F+G) or not (A+B+C+D+E+H) when having sickness benefits or disability pension was used as the outcome measure.

The associations between independent variables and outcome measure were calculated with binary logistic regression analysis using IBM SPSS Statistics version 20. The adequacy of the models was evaluated with goodness-of-fit statistics based on Hosmer-Lemeshow decile-of-risk test [49,50]; a model was rejected if p<0.05. The results are presented as crude and multivariate odds ratios (ORs) with 95% confidence intervals (CIs). Most analyses were stratified by gender due to the large gender differences in rates of hearing difficulties [3,28,37], sick leave [32,51-53], and unemployment [51].

The Regional Ethics Board of Stockholm, Sweden approved the study.

## Results

Table 1 shows basic characteristics of the total study population, of those with self-reported hearing difficulties, and of those with sickness benefits or disability pension as main income source.

**Table 1:** Basic characteristics of the study population (n=19 045)

	**Men**			**Women**		
	**Total**	**With hearing difficulties**	**With sickness benefits or disability pension**	**Total**	**With hearing difficulties**	**With sickness benefits or disability pension**
**Variable**	**n (% of total men)**	**n (% of total men within row)**	**n (% of total men within row)**	**n (% of total women)**	**n (% of total women within row)**	**n (% of total women within row)**
**Gender**	9 287 (100.0)	1 213 (13.1)	776 (8.4)	9 758 (100.0)	956 (9.8)	1 359 (13.9)
**Age (years)**						
** 20-24**	963 (10.4)	51 (5.3)	8 (0.8)	915 (9.4)	50 (5.5)	21 (2.3)
** 25-34**	1 935 (20.8)	140 (7.2)	37 (1.9)	1 951 (20.0)	110 (5.6)	81 (4.2)
** 35-44**	2 245 (24.2)	221 (9.8)	101 (4.5)	2 445 (25.1)	186 (7.6)	243 (9.9)
** 45-54**	2 104 (22.7)	292(13.9)	205 (9.7)	2 264 (23.2)	283 (12.5)	393 (17.4)
** 55-64**	2 040 (22.0)	509 (25.0)	425 (20.8)	2 183 (22.4)	327 (15.0)	621 (28.4)
**Socio-economic status**						
** Professional work level**	1 416 (15.2)	123 (8.7)	43 (3.0)	1 205 (12.3)	81 (6.7)	88 (7.3)
** Intermediate non-manual work level**	1 704 (18.3)	204 (12.0)	92 (5.4)	2 236 (22.9)	193 (8.6)	228 (10.2)
** Assistant non-manual work level**	819 (8.8)	90 (11.0)	81 (9.9)	1 647 (16.9)	165 (10.0)	270 (16.4)
** Self-employed non-professional work level**	996 (10.7)	142 (14.3)	91 (9.1)	475 (4.9)	37 (7.8)	48 (10.1)
** Skilled manual work level**	1 899 (20.4)	318 (16.7)	219 (11.5)	1 361 (13.9)	150 (11.0)	231 (17.0)
** Unskilled/semiskilled manual work level**	1 949 (21.0)	302 (15.5)	243 (12.5)	2 139 (21.9)	283 (13.2)	486 (22.7)
** Students**	504 (5.4)	34 (6.7)	7 (1.4)	695 (7.1)	47 (6.8)	8 (1.2)
**Type of living area**						
** Urban**	3 220 (34.7)	342 (10.6)	210 (6.5)	3 553 (36.4)	293 (8.2)	378 (10.6)
** Semi-urban**	3 365 (36.2)	453 (13.5)	269 (8.0)	3 499 (35.9)	349 (10.0)	491 (14.0)
** Sparsely populated**	2 702 (29.1)	418 (15.5)	297 (11.0)	2 706 (27.7)	314 (11.6)	490 (18.1)
**Country of birth**						
** Sweden**	8 226 (88.6)	1 078 (13.1)	646 (7.9)	8 529 (87.4)	828 (9.7)	1 149 (13.5)
** Elsewhere**	1 061 (11.4)	135 (12.7)	130 (12.3)	1 229 (12.6)	128 (10.4)	210 (17.1)
**Main income source**						
** Work**	7 355 (79.2)	885 (12.0)	0 (0)	6 949 (71.2)	589 (8.5)	0 (0)
** Parental benefits**	19 (0.2)	3 (15.8)	0 (0)	276 (2.8)	14 (5.1)	0 (0)
** Student allowance**	230 (2.5)	15 (6.5)	0 (0)	327 (3.4)	22 (6.7)	0 (0)
** Social assistance**	42 (0.5)	5 (11.9)	0 (0)	44 (0.5)	4 (9.1)	0 (0)
** Unemployment benefits**	495 (5.3)	76 (15.4)	0 (0)	393 (4.0)	54 (13.7)	0 (0)
** Sickness benefits**	228 (2.5)	45 (19.7)	228 (100.0)	424 (4.3)	61 (14.4)	424 (100.0)
** Disability pension**	548 (5.9)	146 (26.6)	548 (100.0)	935 (9.6)	184 (19.7)	935 (100.0)
** Miscellaneous**	370 (4.0)	38 (10.3)	0 (0)	410 (4.2)	28 (6.8)	0 (0)

The prevalence of hearing difficulties was 13.1% in men and 9.8% in women. Among the youngest (20-24-year-olds), about 1 in 20 subjects had hearing difficulties, regardless of gender. The prevalence was higher among men in all other age groups and increased with age to a maximum of 25.0% in men and 15.0% in women in the 55-64-year-olds.

Among the SES categories, hearing difficulties were most common among members of the categories skilled and unskilled/semiskilled manual work level, with a prevalence of 16.7% and 15.5%, respectively, in men, and of 11.0% and 13.2%, respectively, in women.

People living outside metropolitan areas more often reported hearing difficulties than urban dwellers, with the highest prevalence found in sparsely populated areas (15.5% in men and 11.6% in women).

Most people had work as main income source (79.2% in men and 71.2% in women), and 12.0% of those men and 8.5% of those women reported hearing difficulties. In men with either sickness benefits or disability pension as main income source (8.4%), the prevalence of hearing difficulties was 19.7% and 26.6%, respectively. The corresponding figures in women (13.9%) were 14.4% and 19.7%, respectively. Having sickness benefits or disability pension was highly associated with age. Among the SES categories, it was most common among members of the category unskilled/semiskilled manual work level, with a prevalence of 12.5% in men and 22.7% in women. Moreover, it was more common outside metropolitan areas than among urban citizens; the highest prevalence was found among people living in sparsely populated areas (11.0% in men and 18.1% in women). Finally, among those born elsewhere, 12.3% of the men and 17.1% of the women had sickness benefits or disability pension as main income source, which were more frequent than among people born in Sweden.

### Self-reported hearing difficulties as outcome

**Figure 1:**
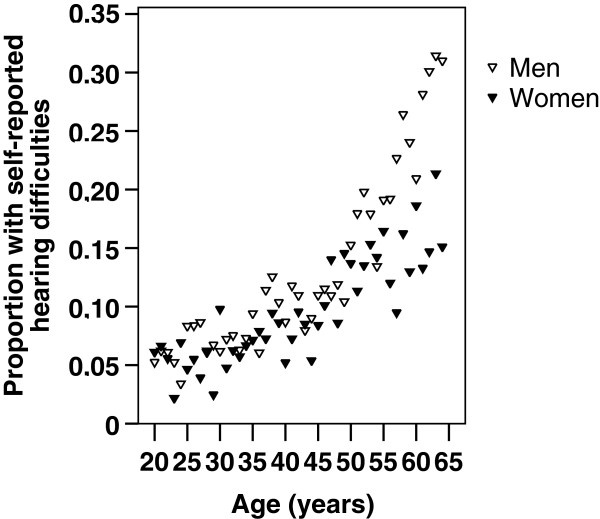
**Proportion of men (n=9 287) and women (n=9 758) in different ages with self-reported hearing difficulties** .

The prevalence of hearing difficulties in different ages is illustrated in Figure 1. Hearing difficulties were significantly more common in men and increased with age; the crude OR of having hearing difficulties was 1.38 (95% CI: 1.26-1.51) for men to women and adjustment with square of age resulted in an OR of 1.42 (1.30-1.56).

From Figure 1 it is clear that the impact of age on hearing difficulties differed between genders. Separate models for men and women were elaborated, which showed a larger difference in the oldest age group; in 55–64 year old men, the crude OR of having hearing difficulties was 5.95 (4.41-8.02), using 20-24-year-olds as reference (model 1; Table 2), whereas in women, the corresponding crude OR was 3.05 (2.24-4.15; model 1; Table 3).

**Table 2:** Odds ratios (ORs) of having hearing difficulties in men (n=9 287)

	**Model 1: Crude**			**Model 2: Age-adjusted**^**a**^		**Model 3: Age- and SES-adjusted**^**a**^		**Model 4: Full factorial**^**a,b**^
**Independent variable**		**OR (95% CI)**		**OR (95% CI)**		**OR (95% CI)**		**OR (95% CI)**
**Age (years)**								
** 20-24**		1				1		1
** 25-34**		1.39 (1.00-1.94)				1.60 (1.14-2.24)		1.59 (1.13-2.24)
** 35-44**		1.95 (1.43-2.67)				2.33 (1.67-3.24)		2.28 (1.63-3.19)
** 45-54**		2.88 (2.12-3.92)				3.41 (2.46-4.73)		3.28 (2.35-4.56)
** 55-64**		5.95 (4.41-8.02)				7.08 (5.14-9.75)		6.54 (4.72-9.05)
**SES**								
** Professional work level**		1		1				1
** Intermediate non-manual work level**		1.43 (1.13-1.81)		1.43 (1.13-1.82)				1.39 (1.09-1.76)
** Assistant non-manual work level**		1.30 (0.97-1.73)		1.34 (1.00-1.79)				1.25 (0.93-1.67)
** Self-employed non-professional work level**		1.75 (1.35-2.26)		1.59 (1.23-2.07)				1.51 (1.16-1.96)
** Skilled manual work level**		2.11 (1.70-2.64)		2.38 (1.90-2.99)				2.17 (1.73-2.74)
** Unskilled/semiskilled manual work level**		1.93 (1.54-2.41)		2.19 (1.75-2.75)				1.98 (1.57-2.50)
** Students**		0.76 (0.51-1.13)		1.84 (1.21-2.82)				1.66 (1.05-2.61)
**Type of living area**								
** Urban**		1		1		1		1
** Semi-urban**		1.31 (1.13-1.52)		1.29 (1.11-1.50)		1.19 (1.02-1.39)		1.18 (1.01-1.38)
** Sparsely populated**		1.54 (1.32-1.79)		1.41 (1.20-1.65)		1.23 (1.05-1.44)		1.22 (1.03-1.43)
**Country of birth**								
** Sweden**		1		1		1		1
** Elsewhere**		0.97 (0.80-1.17)		1.01 (0.83-1.23)		0.95 (0.78-1.16)		0.96 (0.79-1.18)
**Main income source**								
** Work**		1		1		1		1
** Unemployment benefits**		1.33 (1.03-1.71)		1.34 (1.03-1.73)		1.23 (0.94-1.60)		1.22 (0.94-1.59)
** Sickness benefits/disability pension**		2.39 (2.00-2.85)		1.55 (1.29-1.87)		1.36 (1.13-1.65)		1.36 (1.12-1.65)
** Other**		0.74 (0.57-0.98)		1.08 (0.81-1.43)		1.07 (0.78-1.45)		1.09 (0.80-1.48)

^a^Age included as a categorical variable. ^b^Included independent variables are age, socio-economic status (SES), type of living area, country of birth, and main income source.

**Table 3:** Odds ratios (ORs) of having hearing difficulties in women (n=9 758)

		**Model 1: Crude**^**a**^		**Model 2: Age-adjusted**^**a**^		**Model 3: Age- and SES-adjusted**^**a**^		**Model 4: Full factorial**^**a,b**^
**Independent variable**		**OR (95% CI)**		**OR (95% CI)**		**OR (95% CI)**		**OR (95% CI)**
**Age (years)**								
** 20-24**		1				1		1
** 25-34**		1.03 (0.73-1.46)				1.22 (0.86-1.74)		1.19 (0.83-1.70)
** 35-44**		1.42 (1.03-1.97)				1.73 (1.23-2.43)		1.58 (1.12-2.23)
** 45-54**		2.47 (1.81-3.37)				3.01 (2.15-4.20)		2.63 (1.87-3.70)
** 55-64**		3.05 (2.24-4.15)				3.64 (2.61-5.08)		2.99 (2.13-4.20)
**SES**								
** Professional work level**		1		1				1
** Intermediate non-manual work level**		1.31 (1.00-1.72)		1.30 (0.99-1.71)				1.26 (0.96-1.65)
** Assistant non-manual work level**		1.54 (1.17-2.04)		1.48 (1.12-1.95)				1.35 (1.02-1.79)
** Self-employed non-professional work level**		1.17 (0.78-1.76)		1.06 (0.71-1.60)				1.04 (0.69-1.57)
** Skilled manual work level**		1.72 (1.30-2.28)		1.76 (1.33-2.34)				1.58 (1.19-2.12)
** Unskilled/semiskilled manual work level**		2.12 (1.63-2.74)		2.20 (1.70-2.86)				1.88 (1.43-2.45)
** Students**		1.01 (0.69-1.46)		1.82 (1.22-2.70)				1.71 (1.12-2.61)
**Type of living area**								
** Urban**		1		1		1		1
** Semi-urban**		1.23 (1.05-1.45)		1.18 (1.00-1.40)		1.11 (0.94-1.31)		1.09 (0.92-1.29)
** Sparsely populated**		1.46 (1.24-1.73)		1.35 (1.14-1.59)		1.21 (1.01-1.43)		1.18 (0.99-1.40)
**Country of birth**								
** Sweden**		1		1		1		1
** Elsewhere**		1.08 (0.89-1.32)		1.08 (0.89-1.32)		1.01 (0.83-1.24)		1.00 (0.82-1.23)
**Main income source**								
** Work**		1		1		1		1
** Unemployment benefits**		1.72 (1.28-2.32)		1.74 (1.28-2.35)		1.59 (1.17-2.16)		1.58 (1.16-2.14)
** Sickness benefits/disability pension**		2.37 (2.02-2.79)		1.92 (1.63-2.27)		1.73 (1.46-2.06)		1.72 (1.45-2.05)
** Other**		0.74 (0.57-0.96)		1.01 (0.77-1.33)		0.95 (0.71-1.27)		0.95 (0.71-1.28)

^a^Age included as a categorical variable. ^b^Included independent variables are age, socio-economic status (SES), type of living area, country of birth, and main income source.

SES was also significantly associated with hearing difficulties (models 1 and 2; Tables 2 and 3). When adjusting for age, the association was strongest for members of the SES categories skilled and unskilled/semiskilled manual work level with an OR of 2.38 (1.90-2.99) and 2.19 (1.75-2.75), respectively, in men, and of 1.76 (1.33-2.34) and 2.20 (1.70-2.86), respectively, in women, using professional work level as reference.

Hearing difficulties were slightly associated with type of living area, also after age and SES adjustment, whereas no associations were found with country of birth (models 1, 2, and 3; Tables 2 and 3).

**Figure 2:**
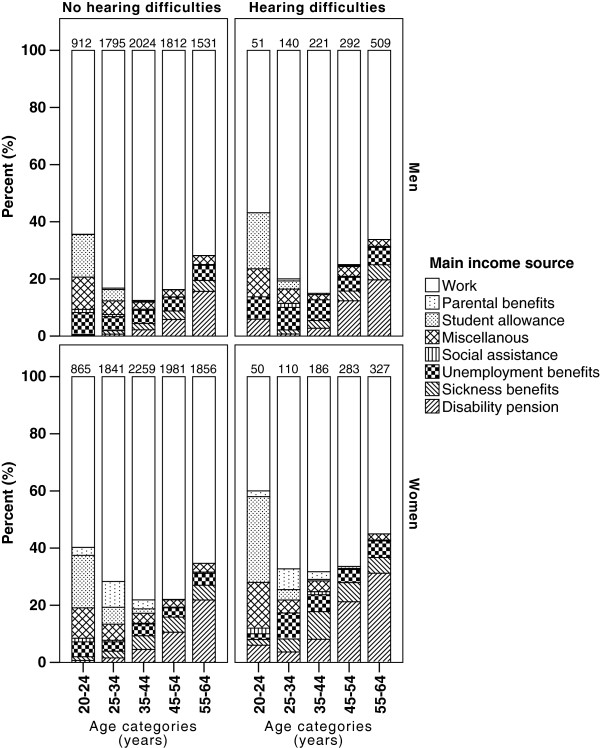
**Main income source of men and women with and without self-reported hearing difficulties in different age strata.** The total number of subjects per bar is indicated at the top of each bar.

The importance of age on type of income source of men and women with and without hearing difficulties is illustrated in Figure 2. After age and SES adjustment, a significant association was found with having sickness benefits or disability pension in men (1.36 (1.13-1.65); reference group: work; model 3; Table 2). In women, significant associations were found with having sickness benefits or disability pension as well as unemployment (1.73 (1.46-2.06) and 1.59 (1.17-2.16), respectively; model 3; Table 3).

Full factorial models including age, SES, type of living area, country of birth, and main income source as independent variables are presented in Tables 2 and 3 (model 4) for men and women, respectively. A full factorial model including both genders is given in Table 4. Crude and multivariate models using age as a continuous variable were also elaborated, with similar results (data not shown).

**Table 4:** Odds ratios (ORs) of having hearing difficulties in men and women (n=19 045)

		**Full factorial**^**a**^	
**Independent variable**		**OR (95% CI)**	
**Age (years)**			
** 20-24**		1	
** 25-34**		1.40 (1.09-1.79)	
** 35-44**		1.95 (1.53-2.47)	
** 45-54**		3.00 (2.37-3.81)	
** 55-64**		4.63 (3.66-5.85)	
**SES**			
** Professional work level**		1	
** Intermediate non-manual work level**		1.33 (1.11-1.59)	
** Assistant non-manual work level**		1.33 (1.09-1.62)	
** Self-employed non-professional work level**		1.36 (1.09-1.69)	
** Skilled manual work level**		1.91 (1.60-2.28)	
** Unskilled/semiskilled manual work level**		1.93 (1.62-2.30)	
** Students**		1.73 (1.27-2.35)	
**Type of living area**			
** Urban**		1	
** Semi-urban**		1.14 (1.02-1.28)	
** Sparsely populated**		1.20 (1.07-1.35)	
**Country of birth**			
** Sweden**		1	
** Elsewhere**		0.98 (0.85-1.13)	
**Main income source**			
** Work**		1	
** Unemployment benefits**		1.35 (1.11-1.66)	
** Sickness benefits/disability pension**		1.54 (1.36-1.75)	
** Other**		1.02 (0.82-1.26)	
**Gender**			
** Women**		1	
** Men**		1.44 (1.31-1.59)	

^a^Included independent variables are age (categorical), socio-economic status (SES), type of living area, country of birth, main income source, and gender.

### Sickness benefits or disability pension as outcome

The characteristics of men and women with sickness benefits or disability pension as compared to those with another main income source were investigated. As shown in Table 1 and Figure 2, having sickness benefits or disability pension was strongly associated with age and gender. There were also strong associations between sickness benefits or disability pension and SES, also after age adjustment (models 1 and 2; Tables 5 and 6).

**Table 5:** Odds ratios (ORs) of having sickness benefits or disability pension in men (n=9 287)

		**Model 1:** ** Crude**^**a**^		**Model 2: ** **Age-adjusted**^**a**^		**Model 3: ** **Age- and SES-adjusted**^**a**^		**Model 4:** ** Full factorial**^**a,b**^
**Independent variable**		**OR (95% CI)**		**OR (95% CI)**		**OR (95% CI)**		**OR (95% CI)**
**Age (years)**								
** 20-24**		1				1		1
** 25-34**		2.33 (1.08-5.02)				2.75 (1.27-5.97)		2.65 (1.22-5.75)
** 35-44**		5.62 (2.73-11.60)				7.06 (3.38-14.75)		6.57 (3.15-13.71)
** 45-54**		12.89 (6.33-26.23)				15.99 (7.74-33.04)		14.45 (7.00-29.83)
** 55-64**		31.41 (15.54-63.51)				39.77 (19.36-81.68)		35.88 (17.47-73.67)
**SES**								
** Professional work level**		1		1				1
** Intermediate non-manual work level**		1.82 (1.26-2.64)		1.82 (1.25-2.64)				1.80 (1.23-2.62)
** Assistant non-manual work level**		3.50 (2.40-5.13)		3.78 (2.56-5.58)				3.68 (2.49-5.44)
** Self-employed non-professional work level**		3.21 (2.21-4.66)		2.83 (1.94-4.13)				2.68 (1.83-3.92)
** Skilled manual work level**		4.16 (2.98-5.82)		5.06 (3.59-7.11)				4.57 (3.23-6.45)
** Unskilled/semiskilled manual work level**		4.55 (3.26-6.34)		5.71 (4.07-8.01)				5.16 (3.66-7.28)
** Students**		0.45 (0.20-1.01)		2.45 (1.06-5.68)				2.02 (0.87-4.70)
**Self-reported hearing difficulties**								
** No**		1		1		1		1
** Yes**		2.39 (2.01-2.85)		1.52 (1.26-1.82)		1.36 (1.12-1.64)		1.36 (1.13-1.64)
**Type of living area**								
** Urban**		1		1		1		1
** Semi-urban**		1.25 (1.03-1.50)		1.21 (1.00-1.47)		1.03 (0.85-1.26)		1.07 (0.88-1.31)
** Sparsely populated**		1.77 (1.47-2.13)		1.55 (1.28-1.87)		1.20 (0.98-1.46)		1.29 (1.06-1.58)
**Country of birth**								
** Sweden**		1		1		1		1
** Elsewhere**		1.64 (1.34-2.00)		1.85 (1.50-2.28)		1.71 (1.37-2.12)		1.81 (1.45-2.25)

^a^Age included as a categorical variable. ^b^Included independent variables are age, socio-economic status (SES), self-reported hearing difficulties, type of living area, and country of birth.

**Table 6:** Odds ratios (ORs) of having sickness benefits or disability pension in women (n=9 758)

		**Model 1: ** **Crude**^**a**^		**Model 2:** ** Age-adjusted**^**a**^		**Model 3: ** **Age- and SES-adjusted**^**a**^		**Model 4:** ** Full factorial**^**a,b**^
**Independent variable**		**OR (95% CI)**		**OR (95% CI)**		**OR (95% CI)**		**OR (95% CI)**
**Age (years)**								
** 20-24**		1				1		1
** 25-34**		1.84 (1.13-3.00)				1.98 (1.21-3.23)		1.95 (1.19-3.18)
** 35-44**		4.70 (2.99-7.39)				4.90 (3.09-7.76)		4.62 (2.92-7.32)
** 45-54**		8.94 (5.72-13.97)				9.13 (5.79-14.38)		8.29 (5.26-13.07)
** 55-64**		16.93 (10.87-26.35)				16.76 (10.67-26.33)		15.32 (9.75-24.08)
**SES**								
** Professional work level**		1		1				1
** Intermediate non-manual work level**		1.44 (1.12-1.86)		1.44 (1.11-1.86)				1.38 (1.06-1.80)
** Assistant non-manual work level**		2.49 (1.93-3.21)		2.38 (1.83-3.08)				2.27 (1.75-2.95)
** Self-employed non-professional work level**		1.43 (0.99-2.06)		1.24 (0.85-1.80)				1.18 (0.81-1.72)
** Skilled manual work level**		2.59 (2.00-3.36)		2.83 (2.17-3.68)				2.55 (1.95-3.33)
** Unskilled/semiskilled manual work level**		3.73 (2.94-4.74)		4.27 (3.34-5.46)				3.74 (2.91-4.80)
** Students**		0.15 (0.07-0.31)		0.47 (0.22-0.99)				0.41 (0.19-0.87)
**Self-reported hearing difficulties**								
** No**		1		1		1		1
** Yes**		2.38 (2.03-2.79)		1.86 (1.57-2.19)		1.70 (1.43-2.01)		1.68 (1.42-1.99)
**Type of living area**								
** Urban**		1		1		1		1
** Semi-urban**		1.37 (1.19-1.58)		1.30 (1.12-1.50)		1.15 (0.99-1.34)		1.17 (1.01-1.37)
** Sparsely populated**		1.86 (1.61-2.15)		1.65 (1.42-1.92)		1.34 (1.15-1.57)		1.39 (1.19-1.63)
**Country of birth**								
** Sweden**		1		1		1		1
** Elsewhere**		1.32 (1.13-1.55)		1.39 (1.17-1.64)		1.29 (1.08-1.53)		1.37 (1.15-1.63)

^a^Age included as a categorical variable. ^b^Included independent variables are age, socio-economic status (SES), self-reported hearing difficulties, type of living area, and country of birth.

The unadjusted OR of having sickness benefits or disability pension was 2.39 (2.01-2.85) and 2.38 (2.03-2.79) in men and women with hearing difficulties, respectively (model 1; Tables 5 and 6, respectively). After age and SES adjustment, the corresponding ORs decreased somewhat, to 1.36 (1.12-1.64) in men and 1.70 (1.43-2.01) in women (model 3; Tables 5 and 6).

Full factorial models including age, SES, hearing difficulties, type of living area, and country of birth as independent variables are presented in Tables 5 and 6 (model 4) for men and women, respectively. Crude and multivariate models with age as a continuous variable were also elaborated, with similar results (data not shown).

## Discussion

This cross-sectional population-based study including 19 045 subjects suggests that people with hearing difficulties are more likely to be dependent on unemployment benefits, sickness benefits, or disability pension than their normal-hearing counterparts. Hearing difficulties were more common in men, but after adjustment with age and SES as well as with type of living area and country of birth, a significant association with long-term unemployment was found only in women, and the associations with long-term sickness absence and disability pension tended to be stronger in women.

The associations between having hearing difficulties and unemployment, sickness absence, and disability pension presented here are in agreement with previous results [25,26,28-30,32-34]. Moreover, this investigation shows that significant associations remain when adjusting for SES, type of living region, and country of birth and not only for gender and age.

Causality cannot be derived from a cross-sectional study, so one can only speculate about the reasons for the associations found in the present investigation. Hearing difficulties has previously been associated with poorer health [14,31,54], work-related stress [14,30,31,54], and work-related accidents [26,55-57], which may eventually lead to sickness absence [30,33] and disability pension [25,33,34]. On the other hand, transitions from paid employment to unemployment, long-term sick leave, and even maternal leave have been associated with increased psychological distress [58], which may cause health problems, including hearing difficulties [24]. Moreover, people on long-term sick leave or disability pension are more likely to have other somatic complaints, which increases the likelihood of reporting hearing difficulties [23], presumably in part due to a reduced ability to cope with the hearing problems or due to a higher awareness of symptoms of bad health.

Women with hearing difficulties are often found to be worse off than their male counterparts [28,31,34,54], which is in line with our results. Women seem to perceive their hearing impairment as being more negative than men do [59,60], possibly because the disability is generally associated with men [61] and affects skills traditionally associated with women, namely communication and nurturing roles [62].

Hearing impairment may have a negative impact on educational performance [25-27]. On the other hand, lower education may result in a more noisy work environment, thus increasing the risk of acquired hearing difficulties [36,38]. It has also been found that hearing-impaired individuals perceive the levels of background noise as being higher than their normal-hearing colleagues do [30], and high noise exposure may in itself increase the risk of work-related accidents [55-57,63], distress symptoms [63], and sickness absence [63]. In the present investigation, these issues were dealt with by adjusting the results with SES. It was found that hearing difficulties were more common among subjects of manual work level than of non-manual work level, in accordance with previous studies [35-37]. However, in one of these earlier studies, which used PTA for hearing assessment, it was questioned whether women’s occupational class is a suitable indicator for socio-economic position in health matters since the researchers found no associations with occupational class in women [35], thus in contrast to the results presented in this paper. Most likely, the discrepancies are partly caused by differences in how hearing difficulties are measured. Moreover, more research is needed on the impact of gender on the results of PTA and self-reported hearing measures.

The prevalence of hearing difficulties in the present investigation was 13.1% in men and 9.8% in women, in well agreement with a new Swedish study on 16-64-year-olds, in which 14.1% of the men and 10.2% of the women reported hearing difficulties [64]. The age-specific prevalence of self-reported hearing difficulties in women in Sweden was recently shown to be slightly higher than in the present investigation (10.2% in 35-44-, 13.5% in 45-54-, and 18.1% in 55-64-year-olds (our study: 7.6%, 12.5%, and 15.0%, respectively)) [65], possibly due to differences in subjects included or how the question on self-reported hearing difficulties was formulated. The fact that there was a higher proportion of men than women with hearing difficulties in all ages but the youngest is in accordance with earlier results [37,64]. Differential estrogen exposures have been suggested as a cause for the higher prevalence of hearing difficulties in men [64]. Another likely explanation is the disparate occupational environments of working men and women [51].

In men, small regional differences in the prevalence of hearing difficulties were found when adjusting for age and SES as well as for all studied confounders; men living outside metropolitan areas were more likely to report hearing difficulties than urban dwellers. This is in line with the results of two other studies from Sweden [4,37]. Possibly, these men are more exposed to spare time noise than women and urban dwellers are. Such noisy, traditionally male, leisure activities are shoot hunting, use of noisy tools, and driving noisy vehicles, e.g. snowmobiles. Another suggestion is that men living outside urban areas have noisier jobs, a difference that the included SES variable fails to completely adjust for. For example, members of the SES categories of manual work level in rural areas may be numerically dominated by wood workers and miners, which are traditionally male and very noisy occupations rarely found in larger cities.

### Strengths and limitations

Benefits of this study are the large sample size and that the population under investigation is representative of the adult population of Sweden. Another strength is the high quality of the data derived from the population-based register LISA. Data on hearing difficulties and SES came from ULF/SILC, a well-evaluated annual survey that has been running for almost 40 years. Yet other strengths are that information on type of income source was available for the same year as participating in the survey and that adjustment was performed with several potentially important variables, i.e. gender, age, SES, type of living region, and country of birth. Nevertheless, the identified associations might be due to confounders not included in the analyses.

As in all surveys, the question about hearing difficulties might have been interpreted in different ways by the participants. Another issue with self-reported health is that some people’s reports may be designed to justify their absence from the labor market [66], which would cause an overestimation of the associations of hearing difficulties with unemployment, sickness benefits, and disability pension. Another possible limitation is that people with severe hearing difficulties might have chosen not to participate in ULF/SILC. In that case, our results are an underestimation. However, people in Sweden with hearing difficulties have for a long time been able to communicate by telephone using telecommunications device for the deaf (TDD), which transmit typed text over regular telephone lines. When appropriate, Statistics Sweden also utilizes video telecommunication through a community service free of charge that offers relay and distance interpretation of the call via a sign language interpreter. A person who uses sign language can access the service via computer, videophone, or 3G.

## Conclusions

This cross-sectional study suggests that men and women with hearing difficulties are more likely to be dependent on unemployment benefits, sickness benefits, or disability pension than their normal-hearing counterparts, also after adjustment for age, SES, type of living area, and country of birth. Future investigations are warranted to explore the causalities of these associations.

Hearing difficulties were more prevalent in men, but a significant association with unemployment was found only in women, and the associations with long-term sickness absence and disability pension tended to be stronger in women. Hitherto, most studies on hearing difficulties have been performed on men, although the increasing prevalence in young individuals has been addressed in several recent studies. The results presented here call for more studies on the situation in hearing-impaired women, a neglected area of research.

## Competing interests

The authors have no competing interests to declare.

## Authors’ contributions

PVP, AW, and KA conceived and designed the experiments. PVP conducted the statistical analyzes and drafted the manuscript together with AF and KA, with essential input from AW. All authors read and approved the final version of the manuscript.

## Pre-publication history

The pre-publication history for this paper can be accessed here:

http://www.biomedcentral.com/1471-2458/12/874/prepub
